# Extracellular Traps at the Crossroads of Immune Evasion and Antimicrobial Resistance: The Case of *Escherichia coli* in the Reproductive Tract

**DOI:** 10.3390/ijms27177608

**Published:** 2026-08-25

**Authors:** Gina Bertolotto, Pamela Uribe, Bárbara Rosales, Raúl Sánchez, Fabiola Zambrano

**Affiliations:** 1 Center of Excellence in Translational Medicine-Scientific and Technological Bioresource Nucleus (CEMT-BIOREN), Faculty of Medicine, Universidad de La Frontera, Temuco 4780000, Chile; gina.bertolotto@ufrontera.cl (G.B.); pamela.uribe@ufrontera.cl (P.U.); b.rosales01@ufromail.cl (B.R.); raul.sanchez@ufrontera.cl (R.S.); 2 Department of Obstetrics and Gynecology, Faculty of Medicine, Universidad de La Frontera, Temuco 4780000, Chile; 3 Department of Internal Medicine, Faculty of Medicine, Universidad de La Frontera, Temuco 4780000, Chile; 4 Department of Preclinical Sciences, Faculty of Medicine, Universidad de La Frontera, Temuco 4780000, Chile

**Keywords:** NETosis, immune evasion, antimicrobial resistance, reproductive tract infections

## Abstract

Extraintestinal pathogenic *Escherichia coli* (ExPEC) is among the bacterial pathogens associated with infections of the female and male reproductive tracts, where it may contribute to inflammatory processes and impaired reproductive function. In this context, neutrophils constitute a critical component of host defense through the release of neutrophil extracellular traps (NETs), web-like structures composed of decondensed chromatin, histones, and antimicrobial proteins that capture and eliminate invading microorganisms. However, the interaction between *E. coli* and NETs is highly dynamic, as the bacterium has evolved immune-evasion mechanisms that facilitate persistence within the host. This raises a critical paradox: NETs may shift from an antimicrobial defense mechanism to a microenvironmental factor that inadvertently favors bacterial persistence and chronic inflammation. This review synthesizes current evidence on the colonization, virulence, and immune-evasion mechanisms of *E. coli* in the reproductive tract, with particular emphasis on the modulation of NETosis, biofilm formation, and their contribution to antimicrobial resistance. We further discuss the dual role of NETs as an innate immune defense mechanism and, conversely, as a potential contributor to tissue injury, oxidative stress, and chronic inflammation when dysregulated. Understanding the interplay among *E. coli*, extracellular traps, and antimicrobial resistance provides a valuable conceptual framework for the development of novel therapeutic approaches aimed at limiting bacterial persistence, preserving reproductive function, and addressing the growing global threat of antimicrobial resistance.

## 1. Introduction

*Escherichia coli* (*E. coli*), a Gram-negative bacterium, typically resides as a commensal member of the human intestinal microbiota. However, certain strains can disseminate beyond the gastrointestinal tract, giving rise to extraintestinal pathogenic *E. coli* (ExPEC), which compromise host tissue integrity and predominantly affect women, older adults, and immunocompromised individuals [1].

Within the female reproductive tract, *E. coli* has been associated with reproductive tract infections and has been used in experimental models of endometritis to investigate bacteria-induced inflammatory responses [2]. In the male reproductive tract, this pathogen is frequently associated with bacterial prostatitis, non-sexually transmitted epididymitis, and bacteriospermia, exerting direct detrimental effects on spermatozoa [3,4]. Furthermore, *E. coli* is a uropathogen frequently associated with ascending acute epididymitis in men of reproductive age, which may be accompanied by a pronounced inflammatory response. The resulting inflammatory microenvironment disrupts essential biochemical processes and compromises sperm function, ultimately contributing to male reproductive dysfunction [5].

*E. coli* has been detected in genital tract infections associated with impaired reproductive outcomes, potentially through mechanisms involving persistent inflammation within the reproductive tract [4]. Upon bacterial invasion, the innate immune system of the uterus and urogenital tract serves as the first line of defense, relying heavily on neutrophil-mediated responses. Neutrophils counteract invading pathogens through the release of neutrophil extracellular traps (NETs), web-like structures composed of decondensed DNA decorated with cytotoxic and antimicrobial proteins that immobilize and eliminate microorganisms [6]. However, excessive or dysregulated NET formation may induce immunopathological damage to the reproductive epithelium and contribute to the persistence of inflammatory conditions such as endometritis [7].

Concurrently, the ability of *E. coli* to evade NET-mediated killing through nuclease production, biofilm formation, and resistance to antimicrobial peptides promotes bacterial persistence and complicates pathogen clearance [8]. Thus, NET formation should not be viewed as synonymous with effective bacterial clearance. Antimicrobial resistance in *E. coli* has emerged as a major global public health concern. The increasing prevalence of strains resistant to β-lactams, fluoroquinolones, and other antimicrobial agents restricts available therapeutic options and favors the establishment of persistent infections [9]. Importantly, persistence and antimicrobial resistance are distinct but potentially reinforcing phenomena. Alarmingly, antimicrobial resistance is projected to become one of the most significant global health challenges by 2050 [1]. Furthermore, although genital tract infections are widely recognized as an important contributor to human infertility, consensus guidelines for the microbiological management of infertile couples undergoing fertility treatments remain lacking [4]. This gap is particularly relevant when microbial detection does not necessarily distinguish colonization from clinically meaningful infection.

Despite growing interest in extracellular traps and the pathogenic mechanisms of *E. coli* over recent years, the interplay among NETosis, immune evasion, and antimicrobial resistance within the reproductive tract remains incompletely understood. A key unresolved question is whether NETs primarily restrict *E. coli* infection or, under persistent conditions, inadvertently contribute to a microenvironment permissive to bacterial survival. Therefore, this review aims to critically examine the current evidence regarding the role of extracellular traps in reproductive tract defense and to discuss the mechanisms by which *E. coli* circumvents these immune responses, thereby promoting antimicrobial resistance, bacterial persistence, and fertility impairment.

## 2. *Escherichia coli* in the Reproductive Tract

Commensal intestinal strains of *E. coli* rarely cause disease. However, a subset of these bacteria has acquired specific virulence traits that enhance their ability to adapt to extraintestinal niches and cause a broad spectrum of infections [8]. Bacterial infections affecting both the female and male reproductive tracts are recognized as important contributors to infertility [4].

Infertility is defined as the failure to achieve conception after at least one year of regular, unprotected sexual intercourse [10]. Its underlying causes may arise from pathological conditions affecting either or both partners [11]. Among the microorganisms implicated in genital tract infections, *E. coli* and *Staphylococcus aureus* are among the most frequently identified bacterial species [12].

A study conducted at the Centre for Diagnosis and Treatment of Couple Infertility at the University Hospital of Siena evaluated 285 infertile couples for the presence of genital tract pathogens prior to undergoing in vitro fertilization (IVF). Notably, none of the participants exhibited clinical signs or symptoms of genital infection. The most prevalent microorganisms detected among infected couples were *Enterococcus faecalis* (32.6%), *E. coli* (22.0%), and *Streptococcus agalactiae* (20.5%), among others at lower frequencies. A significant association was observed between reduced vaginal *lactobacilli* abundance and the presence of genital tract pathogens in women. Furthermore, IVF success rates were slightly higher in non-infected couples than in infected couples [4]. These findings underscore the clinical relevance of bacterial genital tract infections and their potential impact on reproductive outcomes and fertility.

Collectively, these findings support the clinical relevance of *E. coli* in reproductive tract infections; however, its detection alone does not establish a causal role in infertility. Disease development likely depends on the interplay between bacterial virulence, host immune responses, and the local reproductive microenvironment, which determines whether *E. coli* is cleared or progresses toward persistent infection and inflammation. These interactions are particularly relevant in the female and male reproductive tracts, where *E. coli* has been associated with inflammatory disorders and impaired reproductive function.

### 2.1. Female Reproductive Tract

Bacterial infections of the female reproductive tract are common and may lead to serious reproductive and obstetric complications. The vaginal microbiota can be altered by several factors, including sexual behavior, antimicrobial use, physiological changes during the menstrual cycle, vaginal douching, tampon use, and personal hygiene practices [13]. Certain strains of *E. coli* have been identified as potential causative agents of vaginal infections [14]. However, the detection of *E. coli* does not necessarily imply pathogenicity, as disease likely depends on strain-specific virulence traits and the local host–microbiota interactions. In this context, *E. coli* is considered an opportunistic pathogen and a frequent cause of human disease [15]. Disruptions in vaginal microbiota have been associated with conditions such as endometritis, spontaneous abortion, infertility, and preterm birth [12]. Notably, some of these conditions may develop in the absence of overt clinical symptoms [15].

Female genital tract infections may involve the vagina, cervix, uterus, fallopian tubes, or the surrounding pelvic structures. Ascending infections are considered particularly relevant to infertility because they can result in pelvic inflammatory disease and salpingitis, which may ultimately lead to tissue adhesions and tubal damage [4].

Endometritis is characterized by inflammation of the endometrium, the mucosal lining of the uterine cavity. This condition occurs in both humans and animals and may impair fertility and reproductive performance [7]. The pathogenesis of endometritis is complex and can involve bacterial infections. *E. coli* has been used in experimental models of endometritis to investigate bacteria-induced inflammatory responses. As the first cellular barrier within the uterine cavity, endometrial epithelial cells are among the earliest cells to encounter invading pathogens. These cells play a crucial role in the innate immune defense of the uterus. Consequently, disruption of endometrial epithelial integrity facilitates microbial invasion and infection, triggering the recruitment of neutrophils to the uterine cavity through the endometrium and promoting an early immune response against invading pathogens [7].

Experimental models of *E. coli* infection in the female reproductive tract have shown disruption of endometrial epithelial integrity and recruitment of neutrophils [2]. In murine models, even low-dose *E. coli* inoculation can result in robust vaginal colonization and marked uterine inflammation, providing an experimental framework for investigating the local inflammatory consequences of ascending infection [15]. Genomic signatures of ExPEC in vaginal microbiota have also been linked to reduced *Lactobacillus* abundance and increased obstetric risk, though clinical validation in human infertility cohorts remains an ongoing research priority [4].

Experimental evidence suggests that *E. coli*-associated inflammatory responses may influence reproductive function through alterations in endocrine and inflammatory pathways [7]. The recognition of *E. coli* lipopolysaccharide (LPS) by Toll-like receptor 4 (TLR4) in the endometrium triggers a robust release of pro-inflammatory cytokines, including TNF-α, IL-1β, and IL-6 [16]. These mediators directly interfere with steroidogenesis by inhibiting the expression of key enzymes in granulosa cells and perturbing the hypothalamic–pituitary–ovarian axis, which suppresses the preovulatory surge of luteinizing hormone [17].

Furthermore, experimental evidence in equine models has shown that *E. coli*-induced inflammation alters the synthesis of prostaglandins (PGE2 and PGF2α), which are essential for luteal regulation [18]. This disruption leads to dysfunctional growth and reversion of the corpus luteum, resulting in the delayed return of ovarian cycles and extended luteal phases observed in clinical populations. Together, these inflammatory and endocrine alterations may create an unfavorable uterine environment and potentially impair reproductive function [7].

The consequences of vaginal infections are highly variable, ranging from psychological distress to spontaneous abortion and low birth weight in newborns. Globally, approximately 13 million infants are born prematurely each year, representing nearly 10% of all births, and around 30% of these cases have been associated with infectious processes [14].

### 2.2. Male Reproductive Tract

Several male genital disorders have been associated with impaired fertility, including genital trauma, infections affecting semen, testes, and accessory sex glands, genital tract obstruction, varicocele, congenital genital malformations, endocrine and metabolic disorders, substance use and abuse, and suspected psychiatric disorders [11].

According to a study conducted in infertile men, several microorganisms of the male genital tract, including *Escherichia coli*, *Ureaplasma urealyticum*, and *Staphylococcus aureus*, have been associated with alterations in semen quality [11]. However, microbial detection does not necessarily establish causality, particularly given the potential for asymptomatic colonization of the male genital tract. The presence of these microorganisms may negatively affect different sperm parameters through direct mechanisms, such as interaction with spermatozoa, and indirect mechanisms associated with the induction of oxidative stress and activation of inflammatory responses involving the release of pro-inflammatory cytokines. Although the immune response represents an essential mechanism for controlling infections, persistent immune activation may contribute to sperm damage and impair male reproductive function [19]. Thus, reproductive damage may reflect not only bacterial virulence but also a dysregulated or unresolved host inflammatory response.

Genital infection and inflammation can compromise male fertility through multiple mechanisms, including impaired spermatogenesis, disruption of sperm function, increased production of reactive oxygen species (ROS) leading to DNA fragmentation or oxidative modification of proteins, generation of antisperm antibodies, and obstruction of the seminal tract [4]. The balance between effective bacterial clearance and inflammation-mediated sperm damage may therefore represent a critical determinant of infection-associated male infertility.

*E. coli* is frequently implicated in non-sexually transmitted epididymo-orchitis and bacterial prostatitis and may therefore contribute to impaired male reproductive function [11]. Furthermore, *E. coli* has been shown to induce apoptosis in sperm cells [4].

Interestingly, recent case–control studies have reported a paradoxical prevalence of *E. coli* in semen samples, where the bacterium was found at higher frequencies in fertile men compared to infertile cohorts. However, the detrimental effects on sperm parameters, such as morphology, viability, and motility, were significantly more pronounced in the infertile group, suggesting that host susceptibility and the local immune environment are critical determinants of pathological outcomes [20]. Thus, bacterial prevalence alone may be a poor predictor of reproductive damage.

Clinical data on bacteriospermia indicate that *E. coli* infection is associated with a 30% reduction in sperm motility and a 40% increase in the DNA fragmentation index (DFI). These impairments are strongly correlated with the upregulation of inflammatory cytokines, such as IL-6, TNF-α, and IL-1β, within the seminal plasma, creating a maladaptive environment that impedes sperm function [21]. This suggests that sperm dysfunction may reflect the combined effects of bacterial injury and inflammation-driven damage.

A critical clinical validation of the progression from localized infection to chronic reproductive dysfunction is the formation of tertiary lymphoid organs (TLOs) in the human cauda epididymidis. Following chronic epididymitis, the development of these organized B- and T-cell clusters, which include specialized high endothelial venules (HEVs), indicates a long-term immunological remodeling of the tissue. This pathological process can lead to permanent structural alterations, such as fibrosis and abscess formation, resulting in severely low sperm counts in approximately 40% of affected patients even after antibiotic treatment. Furthermore, the presence of active germinal centers within these TLOs suggests a local production of antibodies that could target spermatozoa, providing a new molecular framework to understand persistent infertility [22]. Importantly, bacterial clearance may therefore not necessarily translate into restoration of reproductive function.

Although most *E. coli* strains are harmless, pathogenic strains have been frequently isolated in male genital tract infections and have been associated with alterations in sperm function and male reproductive health. Several studies have described direct detrimental effects of *E. coli* on sperm motility [23]. This reduction in sperm motility has been attributed to sperm agglutination mediated by bacterial fimbriae, particularly type 1 and P fimbriae. Additionally, *E. coli* has been shown to induce higher ROS production compared with other bacterial species [23].

In support of these findings, a 1996 study evaluated in vitro the effect of a uropathogenic strain of *E. coli* on human sperm motility. The authors observed that the progressive reduction in motility was dependent on bacterial concentration, becoming more pronounced as the number of bacteria increased during incubation. Furthermore, electron microscopy analysis demonstrated the adhesion of *E. coli* to sperm and the associated ultrastructural alterations, providing a possible underlying mechanism for sperm immobilization. These findings suggest that *E. coli* infections in the male reproductive tract can compromise fertility by directly impairing sperm function [24].

A subsequent study further demonstrated that these direct bacterial effects can translate into poor reproductive outcomes in vivo. In a murine model, intravaginal administration of a sperm-agglutinating strain of *E. coli* resulted in temporary infertility, while elimination of the bacteria allowed for the recovery of fertility. These findings suggest that the persistence of *E. coli* in the reproductive tract can reversibly affect reproductive function, underscoring the potential impact of bacteria–sperm interactions on fertility [25]. Collectively, this evidence indicates that the reproductive consequences of *E. coli* infection are determined not merely by bacterial presence but by the balance between direct sperm injury, bacterial persistence, and the magnitude and duration of the host inflammatory response.

## 3. Pathogenesis and Immune Evasion Mechanisms of *E. coli*

Pathogenic *E. coli* strains possess a wide range of virulence factors that enable colonization, persistence, and tissue damage within the host. Many of these determinants are shared among pathogenic *E. coli* strains regardless of the anatomical site of infection [26].

ExPEC strains possess numerous virulence-associated factors, including adhesins, toxins, iron acquisition systems, lipopolysaccharides, polysaccharide capsules, and invasins, which are commonly encoded within pathogenicity islands (PAIs), plasmids, and other mobile genetic elements [27].

Pathogenic *E. coli* infection proceeds via a multistep cascade involving mucosal colonization, evasion of host immune defenses, bacterial proliferation, and ultimately host tissue damage. The remarkable adaptability of these bacteria is largely attributable to their ability to deploy niche-specific virulence factors that facilitate survival and persistence beyond the intestinal environment [28]. Thus, ExPEC pathogenicity may be better understood as a context-dependent deployment of virulence traits rather than as a fixed virulence program. The ability of bacteria to adhere to host cells is a prerequisite for bacterial colonization. This phenomenon, referred to as tissue tropism, involves specific interactions between bacterial adhesins and target receptors expressed on particular host tissues [27]. To colonize the reproductive and urinary tracts, *E. coli* utilizes filamentous surface structures known as fimbriae, which contain adhesins that mediate attachment to epithelial cells. Different fimbrial types are expressed depending on the bacterial strain and the target tissue. Strains colonizing the reproductive tract typically belong to the ExPEC group and share several virulence traits with uropathogenic *E. coli* UPEC strains [28].

Type 1 and P fimbriae mediate adhesion to epithelial cells and therefore represent important colonization factors in extraintestinal infections [27]. Type 1 fimbriae are essential during the early stages of infection, mediating bacterial adhesion through interactions with mannose-containing glycoprotein receptors on epithelial surfaces. In this context, experimental evidence from a mouse model has shown that type 1 fimbriae promote bacterial growth in biofilm-like communities [27]. In contrast, P fimbriae (Pap) promote attachment to specific epithelial cell populations, facilitating bacterial ascension and persistence within deeper tissues [28].

The polysaccharide capsule surrounding the bacterial cell surface can protect *E. coli* against phagocytosis and the bactericidal activity of the immune system. The structural diversity of these capsules may also allow certain *E. coli* strains to mimic host tissue components, thereby reducing their recognition by the immune system [27]. In this context, *E. coli* has evolved multiple strategies to evade host immune responses. Among these, K-antigen capsules function as antiphagocytic shields that reduce immune recognition and enhance bacterial survival by limiting efficient clearance by host defense mechanisms. Furthermore, *E. coli* can modulate fimbrial expression through reversible genetic switching, enabling adaptation to distinct microenvironments while reducing recognition by antibodies and immune cells [28]. Certain strains, particularly uropathogenic isolates, are also capable of forming intracellular bacterial reservoirs and biofilm-like communities within epithelial cells. These intracellular niches protect bacteria from both host immune defenses and antimicrobial therapies, thereby promoting recurrent and persistent infections.

Iron plays a key role in essential cellular processes, including cellular respiration and DNA replication. However, iron availability within the host is tightly restricted, creating a major nutritional challenge for invading bacteria. To overcome this limitation, pathogenic bacteria have evolved specialized iron-acquisition mechanisms that support survival, adaptation, and virulence. Among these, siderophores enable the sequestration of ferric iron (Fe^3+^) from host iron-binding proteins such as ferritin and transferrin. Extraintestinal pathogenic *E. coli* strains may express several siderophores associated with enhanced virulence, including enterobactin and salmochelin (catecholate siderophores), yersiniabactin (a phenolate siderophore), and aerobactin [27]. These molecules enable bacterial growth even under conditions of iron restriction imposed by the host, thereby enhancing fitness, persistence, and pathogenic potential [28]. Importantly, these mechanisms may act cooperatively, allowing *E. coli* to simultaneously evade immune clearance, withstand nutritional restriction, and establish persistent infection.

In a study of UPEC strains isolated from high vaginal swab samples of fertile and infertile women, the virulence genes *sfa*, *afa*, *cnf1*, and *fim* were each detected in 72.72% of the isolates [17]. Certain *E. coli* strains express TcpC, a virulence factor that can interfere with NET formation. TcpC promotes the ubiquitination and degradation of peptidylarginine deiminase 4 (PAD4), thereby reducing histone citrullination and chromatin decondensation and ultimately inhibiting NET formation [16]. This represents a particularly relevant immune-evasion strategy, as it targets the host machinery required for NET formation rather than merely resisting NET-mediated killing.

In addition to protein factors such as TcpC, the O-antigen component of LPS has been implicated in the interaction between *E. coli* and NETs. Experimental evidence indicates that the O-antigen contributes to NET induction while also enhancing bacterial resistance to NET-mediated killing [29]. This dual behavior highlights the complexity of the *E. coli*–NET interaction: bacterial structures may simultaneously trigger NET formation while conferring resistance to its antimicrobial effects.

These immune-evasion mechanisms may contribute to bacterial persistence by reducing effective host clearance. Persistent bacterial populations can subsequently encounter conditions that favor biofilm development and antimicrobial tolerance, although the relationship between immune evasion and antimicrobial resistance is complex and remains under investigation. These interactions are discussed in greater detail in Section 6.

## 4. Innate Immune Response to *E. coli* and the Formation of NETs

Neutrophil extracellular trap (NET) formation is an innate immune mechanism involving the release of decondensed chromatin associated with antimicrobial proteins, which contributes to pathogen immobilization and containment. NET formation involves intracellular events such as ROS production, PAD4 activation, histone citrullination, and chromatin decondensation; however, these mechanisms have been extensively described elsewhere [7,30]. Therefore, this section focuses specifically on the interaction between *E. coli* and NET-mediated neutrophil responses.

During *E. coli* infection, neutrophils are recruited as part of the innate immune response and contribute to bacterial containment through several antimicrobial mechanisms, including the formation of NETs [31]. However, NET induction should not be interpreted as synonymous with effective bacterial clearance. Experimental studies using uropathogenic *E. coli* mutants have shown that the O-antigen component of LPS contributes to NET induction through TLR4-dependent signaling and NADPH oxidase-mediated ROS production. Loss of the O-antigen significantly reduces ROS generation and subsequent NET formation [29]. This suggests that NET formation may reflect active recognition of specific bacterial determinants rather than merely the magnitude of bacterial exposure.

The interaction between *E. coli* and NETs is therefore bidirectional and strain-dependent. While bacterial components such as the O-antigen of LPS can promote NET formation, specific virulence factors, including TcpC, can interfere with this response [16,29]. These findings illustrate how *E. coli* may simultaneously trigger and modulate neutrophil antimicrobial mechanisms. Thus, the biological outcome of NET formation may depend less on the extent of NETosis itself than on the ability of *E. coli* to withstand, evade, or exploit the resulting extracellular environment.

## 5. NET-Mediated Tissue Damage

Since their discovery, NETs have been recognized as potent antimicrobial structures that play a critical role in host defense. However, despite their ability to eliminate invading pathogens, accumulating evidence indicates that NETs may also contribute to tissue injury and disease pathogenesis when their formation becomes excessive or dysregulated [7].

NET formation is triggered by a wide range of infectious agents, including bacteria, fungi, viruses, and parasites, serving as an important mechanism for pathogen containment. Nevertheless, persistent or uncontrolled NET release has been associated with autoimmune disorders and chronic inflammatory diseases [30]. Within the reproductive tract, excessive NET accumulation may compromise tissue integrity and negatively affect fertility. Prolonged inflammatory responses characterized by the infiltration of lymphocytes and plasma cells have been associated with chronic endometrial degeneration. Whether bacteria act as the primary initiators of these pathological processes or represent secondary colonizers of already damaged tissues remains unclear [32]. This distinction is critical, as NET accumulation may represent either a driver of tissue pathology or a consequence of persistent inflammation.

In the male reproductive tract, studies in patients with acute epididymitis have demonstrated that extracellular traps can physically entangle spermatozoa, resulting in a marked reduction in progressive sperm motility [5]. Moreover, NETosis is accompanied by an oxidative burst that increases ROS production, promoting oxidative damage to sperm plasma membranes and contributing to inflammation-associated male infertility [3]. Thus, while NETs are essential for bacterial containment, their excessive release and the cytotoxic molecules they contain may also damage host tissues, exacerbate oxidative stress, and impair reproductive function. Overall, the reproductive consequences of NETosis may depend on the balance between effective pathogen containment and collateral damage to highly vulnerable reproductive cells.

In the female reproductive tract, experimental studies in equine models have demonstrated that neutrophils release NETs in response to uterine pathogens such as *E. coli*. Although NETs function as an effective first line of antimicrobial defense, excessive NET formation and/or reduced NET degradation may contribute to endometrial fibrosis (endometriosis) and impaired endometrial secretory function [18]. Beyond its detrimental effects on the female reproductive tract, pathogenic UPEC strains also impair male reproductive function by compromising sperm structural integrity, inducing nuclear DNA damage, and ultimately abolishing the capacity for in vitro fertilization [33].

Recent experimental evidence has further elucidated the mechanisms underlying NET-mediated tissue damage. In the female reproductive tract, studies using bovine endometrial epithelial cell models have demonstrated that NETs not only function as physical antimicrobial barriers but also induce pyroptosis in endometrial epithelial cells. This process is mediated by activation of the NLRP3 inflammasome and the upregulation of ASC, caspase-1, caspase-4, and GSDMD-N, leading to increased production of the pro-inflammatory cytokines TNF-α, IL-1β, IL-6, and IL-18. Notably, DNase I treatment attenuated these effects, highlighting the direct contribution of NETs to endometrial inflammatory injury [34]. Importantly, these findings suggest that NETs may actively amplify inflammation rather than merely accumulate as a consequence of tissue injury.

Regarding sperm damage, recent confocal microscopy studies have demonstrated that NETs can physically entrap not only spermatozoa but also developing embryos while simultaneously impairing sperm motility, thereby compromising the efficiency of in vitro fertilization (IVF) [35]. These findings suggest that excessive NET formation may impair reproductive success through both mechanical entrapment and functional deterioration of spermatozoa. Collectively, this evidence challenges the conventional view of NETs as exclusively protective structures and positions their persistence and defective clearance as potential determinants of reproductive tissue damage.

## 6. Antimicrobial Resistance and Its Relationship with Bacterial Persistence

The persistence of ExPEC in extraintestinal niches may involve interactions between immune evasion, biofilm-associated survival, and antimicrobial resistance (AMR). Although these processes have often been studied separately, increasing evidence suggests that they may converge to favor bacterial persistence under antimicrobial and immune pressures [36]. Within this framework, NET-evasion mechanisms may further promote persistence by enabling bacterial survival despite sustained neutrophil-mediated pressure.

The persistence of *E. coli* in extraintestinal niches has been associated with capsular structures that can contribute to protection against host immune defenses and may also reduce antimicrobial accessibility. Recent genomic studies have shown that a limited number of capsular types (K1, K5, K52, K2, and K100) account for approximately 58% of multidrug-resistant extraintestinal infections. This association may reflect the contribution of mobile genetic elements that facilitate the co-transfer of capsular virulence determinants and antimicrobial resistance genes [37]. However, such genomic associations should not be interpreted as evidence that capsule production directly confers antimicrobial resistance.

Antimicrobial resistance may arise through intrinsic mechanisms or be acquired through mutation and horizontal gene transfer. Selective pressure imposed by antimicrobial exposure favors the persistence and expansion of resistant bacterial populations. Acquired resistance determinants can disseminate through conjugation, transduction, and transformation, enabling the exchange of resistance genes within bacterial communities [32].

**Figure 1:**
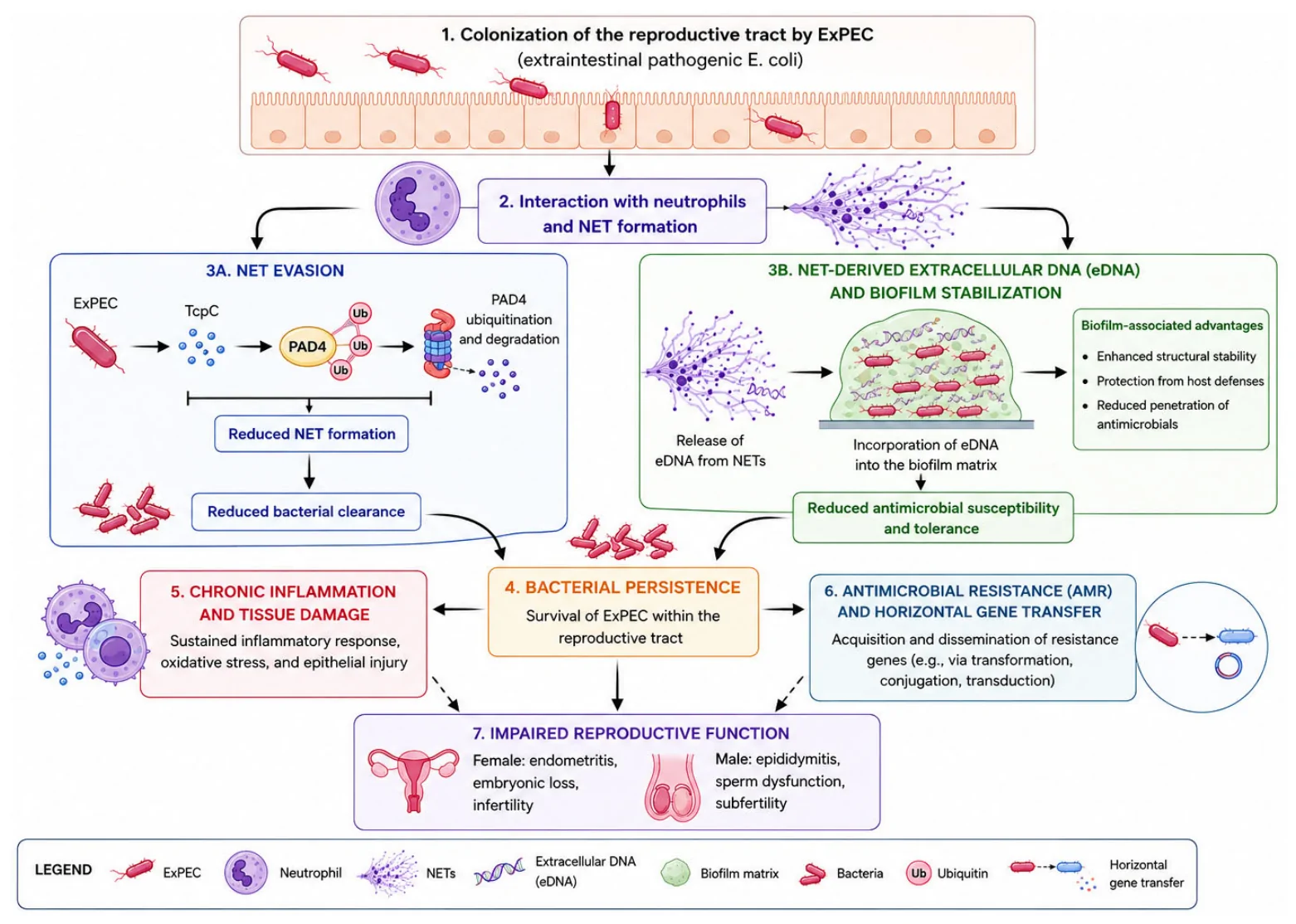
Interplay between neutrophil extracellular traps (NETs), bacterial persistence, biofilm formation, and antimicrobial resistance in extraintestinal pathogenic *Escherichia coli* (ExPEC). Following colonization, ExPEC interacts with neutrophils and NET-mediated antimicrobial responses. Virulence factors such as TcpC can interfere with NET formation through PAD4 ubiquitination and degradation, potentially reducing neutrophil-mediated bacterial clearance. In parallel, extracellular DNA (eDNA) released during NET formation may become incorporated into the biofilm matrix, contributing to biofilm stability and reduced antimicrobial susceptibility. These mechanisms may favor bacterial persistence and sustained inflammatory responses, while biofilm-associated survival and horizontal gene transfer may contribute to the maintenance and dissemination of antimicrobial resistance. Together, these interconnected processes may affect reproductive tissue homeostasis and reproductive function. Solid arrows represent mechanisms supported by current evidence, whereas dashed arrows indicate proposed or incompletely understood relationships that require further investigation. The figure was prepared by the authors with the assistance of generative artificial intelligence tools (ChatGPT-5.6 (OpenAI, San Francisco, CA, USA; https://chatgpt.com/, accessed on 13 July 2026)).

Overall, antimicrobial resistance in extraintestinal *E. coli* reflects the interplay of virulence, immune evasion, biofilm formation, and horizontal gene transfer. NET-derived DNA may further support biofilm formation and bacterial transformation, although its relevance in reproductive tract infections remains unclear. Together, these mechanisms support a model in which immune pressure, bacterial persistence, and antimicrobial selection may interact to sustain difficult-to-eradicate *E. coli* infections, although their specific contribution within the reproductive tract remains to be established. These interconnected mechanisms are summarized in Table 1 and integrated into the conceptual model presented in Figure 1.

Biofilm formation can enhance bacterial persistence by providing a structured extracellular matrix that limits exposure to host immune effectors and antimicrobial agents. Extracellular DNA is an important component of this matrix and may include host-derived DNA released during inflammatory responses, including NET formation. Incorporation of extracellular DNA can contribute to biofilm stability and reduced antimicrobial susceptibility, while the biofilm microenvironment may further favor the persistence of slow-growing bacterial populations [38,39]. Thus, NET-derived DNA may paradoxically shift from a host defense component to a structural resource that favors bacterial persistence.

## 7. Conclusions

Colonization of the reproductive tract by extraintestinal pathogenic *E. coli* (ExPEC) can elicit innate immune responses in which NETs may play a dual role. While NETs contribute to the containment of invading pathogens, excessive or persistent NET formation may promote oxidative stress and inflammatory tissue damage, potentially affecting reproductive tissue integrity and gamete function. Thus, the biological significance of NETs may depend not simply on their formation but on their magnitude, persistence, and timely clearance.

Growing evidence indicates that the interaction between extraintestinal pathogenic *E. coli* and NETs involves multiple mechanisms that may influence bacterial persistence. These include modulation of NET formation through virulence factors such as TcpC and interactions between host-derived extracellular DNA and biofilm development, which may contribute to bacterial persistence and reduced antimicrobial susceptibility. However, whether these mechanisms directly converge in the reproductive tract remains to be demonstrated.

These interconnected processes place NETs at the crossroads of immunity, microbial adaptation, and reproductive dysfunction. A key challenge is therefore to determine when NETs shift from an effective antimicrobial response to a microenvironment that favors inflammation and bacterial persistence. A deeper mechanistic understanding of these interactions could reveal novel therapeutic opportunities aimed at modulating dysregulated NETosis, altering biofilm stability, and enhancing antimicrobial efficacy. Such approaches could not only improve the management of persistent reproductive tract infections but also help preserve fertility. Defining this balance may ultimately be critical for controlling infection without compromising the reproductive functions that innate immunity is intended to protect.

## Figures and Tables

**Table 1:** Virulence factors and host-derived components involved in Escherichia coli immune evasion, NET modulation, antimicrobial resistance, and bacterial persistence.

Virulence Factor/Component	Immune Evasion Mechanism/Effect on NETs	Impact on Antimicrobial Resistance (AMR) and Persistence	References
Type 1 and P Fimbriae	Facilitate epithelial adhesion and colonization, contributing to bacterial persistence within host tissues.	Persistent colonization may favor the establishment of bacterial populations exposed to immune and antimicrobial pressures.	[28,30]
K Antigen Capsule	Functions as an antiphagocytic barrier that limits immune recognition and impairs efficient bacterial clearance.	Capsular types have been associated with multidrug-resistant ExPEC lineages, potentially reflecting linkage between virulence and antimicrobial resistance determinants.	[28,37]
TcpC Protein	Promotes PAD4 ubiquitination and degradation in neutrophils, inhibiting histone citrullination and suppressing NETosis induction.	Inhibition of NET formation may contribute to reduced neutrophil-mediated bacterial clearance and persistence.	[16,36]
NET-Derived Extracellular DNA	Excessive NETosis promotes immunopathology and oxidative stress in the reproductive epithelium.	Extracellular DNA may contribute to biofilm structure, while environmental DNA can serve as genetic material for bacterial transformation. The direct contribution of NET-derived DNA to acquisition of antimicrobial resistance genes remains unclear.	[32]

## Data Availability

No new data were created or analyzed in this study. Data sharing is not applicable to this article.

## Abbreviations

AMR	Antimicrobial Resistance
Ca^2+^	Calcium Ion
ExPEC	Extraintestinal Pathogenic Escherichia coli
HlyA	Hemolysin A
IVF	In Vitro Fertilization
LPS	Lipopolysaccharide
MPO	Myeloperoxidase
NADPH	Nicotinamide Adenine Dinucleotide Phosphate
NE	Neutrophil Elastase
NETs	Neutrophil Extracellular Traps
NETosis	Neutrophil Extracellular Trap Formation
PAD4	Peptidylarginine Deiminase 4
PAMPs	Pathogen-Associated Molecular Patterns
Pap	Pyelonephritis-Associated Pili
PRRs	Pattern Recognition Receptors
ROS	Reactive Oxygen Species
TLR4	Toll-Like Receptor 4
TcpC	Toll/Interleukin-1 Receptor Domain-Containing Protein C
UPEC	Uropathogenic Escherichia coli
